# ZIM1 Combined with Hydrogel Inhibits Senescence of Primary PαS Cells during In Vitro Expansion

**DOI:** 10.3390/ijms24119766

**Published:** 2023-06-05

**Authors:** Yueming Tian, Menglong Hu, Xuenan Liu, Xu Wang, Dazhuang Lu, Zheng Li, Yunsong Liu, Ping Zhang, Yongsheng Zhou

**Affiliations:** 1 Department of Prosthodontics, Peking University School and Hospital of Stomatology, 22 Zhongguancun South Avenue, Haidian District, Beijing 100081, China; tym00007@163.com (Y.T.); menglonghu0611@163.com (M.H.); pkukqlxn@bjmu.edu.cn (X.L.); wangxu0315@bjmu.edu.cn (X.W.); ludazhuang666@gmail.com (D.L.); pkulizheng@126.com (Z.L.); liuyunsong@hsc.pku.edu.cn (Y.L.); 2 National Center for Stomatology, 22 Zhongguancun South Avenue, Haidian District, Beijing 100081, China; 3 National Clinical Research Center for Oral Diseases, 22 Zhongguancun South Avenue, Haidian District, Beijing 100081, China; 4 National Engineering Research Center of Oral Biomaterials and Digital Medical Devices, 22 Zhongguancun South Avenue, Haidian District, Beijing 100081, China; 5 Beijing Key Laboratory of Digital Stomatology, 22 Zhongguancun South Avenue, Haidian District, Beijing 100081, China

**Keywords:** stem cells, cellular senescence, transcriptomics, Wnt pathway, hydrogel

## Abstract

Bone marrow stem cells (BMSCs) are a promising source of seed cells in bone tissue engineering, which needs a great quantity of cells. Cell senescence occurs as they are passaged, which could affect the therapeutic effects of cells. Therefore, this study aims to explore the transcriptomic differences among the uncultured and passaged cells, finding a practical target gene for anti-aging. We sorted PαS (PDGFR-α^+^SCA-1^+^CD45^-^TER119^-^) cells as BMSCs by flow cytometry analysis. The changes in cellular senescence phenotype (Counting Kit-8 (CCK-8) assay, reactive oxygen species (ROS) test, senescence-associated β-galactosidase (SA-β-Gal) activity staining, expression of aging-related genes, telomere-related changes and in vivo differentiation potential) and associated transcriptional alterations during three important cell culture processes (in vivo, first adherence in vitro, first passage, and serial passage in vitro) were studied. Overexpression plasmids of potential target genes were made and examed. Gelatin methacryloyl (GelMA) was applied to explore the anti-aging effects combined with the target gene. Aging-related genes and ROS levels increased, telomerase activity and average telomere length decreased, and SA-β-Gal activities increased as cells were passaged. RNA-seq offered that imprinted zinc-finger gene 1 (*Zim1*) played a critical role in anti-aging during cell culture. Further, *Zim1* combined with GelMA reduced the expression of P16/P53 and ROS levels with doubled telomerase activities. Few SA-β-Gal positive cells were found in the above state. These effects are achieved at least by the activation of Wnt/β-catenin signaling through the regulation of *Wnt2*. The combined application of *Zim1* and hydrogel could inhibit the senescence of BMSCs during in vitro expansion, which may benefit clinical application.

## 1. Introduction

The application of bone marrow stem cells (BMSCs) in regenerative medicine is rapidly progressing, particularly in bone tissue engineering [1,2]. However, BMSCs are a rare group of non-hematopoietic pluripotent cells present in the bone marrow, and their functional characterization and clinical application necessitate extensive ex-vivo manipulation [3,4]. Aging causes stem cells to lose their protective and regenerative capacities, thereby decreasing stem cell numbers and functions [5,6]. BMSCs enter senescence and start to lose their stem cell abilities almost undetectably soon after they are cultured in vitro [7]. Further, the telomere length of Mesenchymal stromal cells (MSCs) decreases during culture expansion [8]. The adipo-osteogenic balance of BMSCs is also dysregulated after long-term in vitro culture [9]. Therefore, regulating BMSC aging is of importance during in vitro culture to promote their clinical translational applications. However, the conventional BMSCs expansion method is to continuously passage culture, which may lead to the high heterogeneity of cell populations or contain hematopoietic cell contamination [10]. These will lead to more frequent medium replacement, more generation and longer culture time [11]. Murine bone marrow-derived PDGFR-α^+^SCA-1^+^CD45^-^TER119^-^ mesenchymal stromal cells (PαS cells), firstly described by Morikawa, had higher proliferative and tri-lineage differentiation capacities, without any hematopoietic potential in vitro [12,13]. PαS cells were therefore considered primary BMSCs, with proliferation and differentiation characteristics of MSCs [13]. Cell status after flow sorting was close to the in vivo situation, which offers the possibility of comparing the biological performance of BMSCs in vivo and in vitro.

Hayflick and Moorhead first proposed that cell senescence is an irreversible and non-divisive state wherein cells retain metabolic activity after they reach their replicative potential in vitro [14]. Senescence is a cellular mechanism wherein dividing cells enter a stable cell generation block under physiological, injury, or stress stimulations [15]. Factors called senescence-associated secretory phenotype (SASP) are released by senescent cells into cell niches. In this state, cells are typically unresponsive to mitogenic or apoptotic signals but remain metabolically active [16,17,18,19]. Most studies use a standard set of markers such as characteristic enlarged and flattened morphology, the presence of multinucleation, stable cell cycle arrest, cytoplasmic DNA increase, chromatin alteration and reorganization, macromolecular damage and metabolic changes, resistance to apoptosis, increased lysosomal compartment, and strong SASP to identify senescence [16,20].

Many hydrogels and polymer crosslinked networks have been designed to simulate the extracellular matrix (ECM) and form 3D constructs suitable for cell culture [21]. These biomaterials have been widely used in biomedical applications such as tissue regeneration, drug delivery, scaffolds, and tissue adhesives [22,23,24,25]. Gelatin methacryloyl (GelMA) is a type of biomaterial synthesized from gelatin and methacrylate. The biocompatibility and special permeability of GelMA make it ideal for cell loading [26]. Low concentrations of GelMA are more conducive to cellular function and growth, with larger pores showing better elasticity and degradation profiles [27,28]. However, there have been few reports on the anti-aging effects of GelMA in PαS cells. GelMA could be useful in the expansion of BMSCs in vitro if its effect on cell senescence is clear.

Compared to human cells and tissue, tissue and cells from mice are easily available, mammalian, and belong to model organisms. The isolation, identification, and detection of BMSCs in mice are, therefore, easier to carry out. In contrast to other non-hematopoietic cells (PDGFR-α^-^SCA-1^-^ cells), PαS cells had higher proliferative and tri-lineage differentiation capacities without any hematopoietic potential in vitro [12,13]. PαS cells were therefore considered primary BMSCs, with proliferation and differentiation characteristics of MSCs [13].

**Figure 1:**
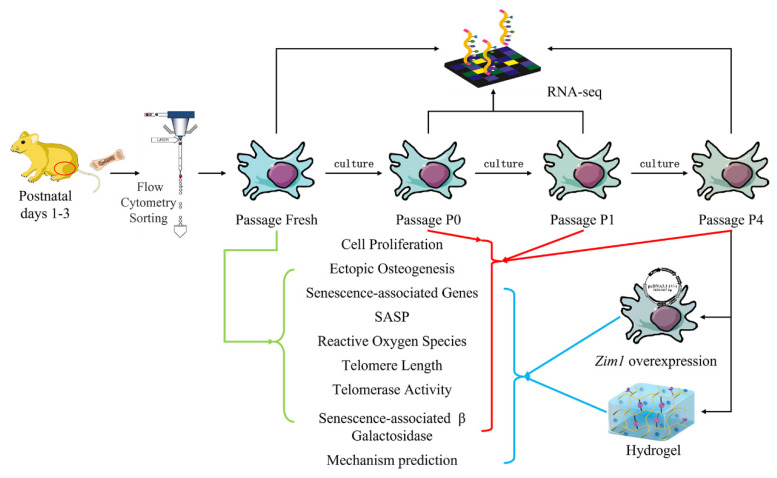
Schematic representation of the study. PαS cells were extracted from the leg bones of mice, and the sorting of PDGFR-α^+^SCA-1^+^CD45^-^TER119^-^ cells was done through flow cytometry. RNA-seq was applied to the cells. Different passages of cells are shown by different colored arrows. Red circle, total limb buds.

The general hypothesis of this study is that the cellular senescence of BMSCs will not change as cells are extracted from in vivo and cultured/passaged in vitro. As shown in Figure 1, cell proliferation, reactive oxygen species test, telomerase and telomere length, senescence β-galactosidase staining, gene expression level, and RNA-seq analysis were applied to verify the hypothesis. The effects of imprinted zinc-finger gene 1 (*Zim1*) combined with GelMA on cellular senescence were also explored.

## 2. Results

### 2.1. Cellular Senescence Increased during the Transition from In Vivo to In Vitro

**Figure 2:**
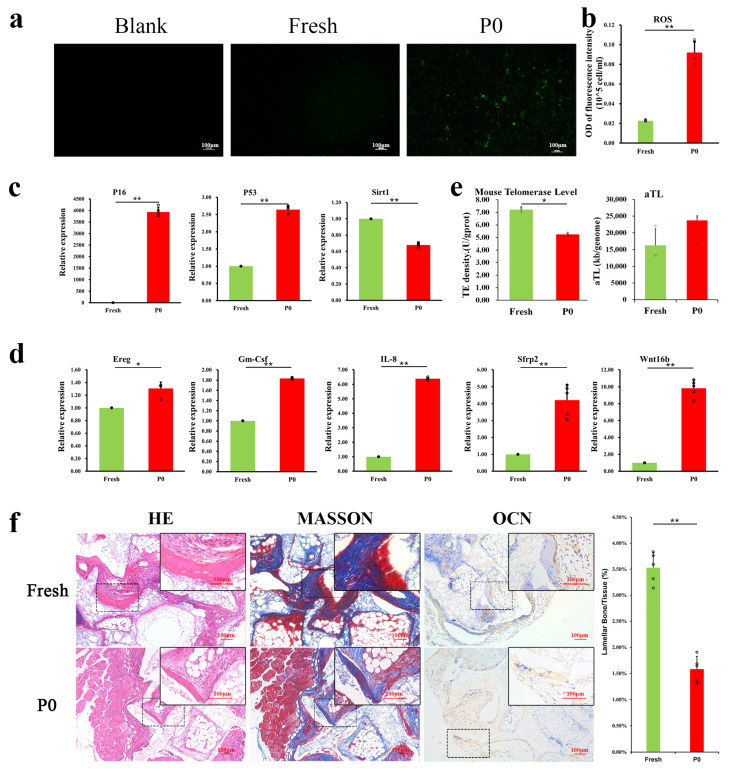
Senescence occurs when PαS cells are cultured from in vivo to in vitro. (**a**) Intracellular total ROS staining of PαS cells between in vivo (Fresh) and in vitro (P0). (**b**) Intracellular total ROS quantitative analysis (*n* = 5) of PαS cells between Fresh and P0 cells. (**c**) qRT-PCR was used to detect the expression of senescence-associated genes (*P16*-left, *P53*-middle, *Sirt1*-right) of different states (passage Fresh and P0) in PαS cells (*n* = 5). (**d**) mRNA levels of SASP genes (*IL-8, Gm-Csf, Sfrp2, Ereg* and *Wnt16b*) of between Fresh and P0 cells (*n* = 5). (**e**) Telomerase activity and average telomere length of different states (passage Fresh and P0) in PαS cells (*n* = 3). (**f**) The ectopic osteogenesis capacity between passage Fresh and P0 (HE-left, Masson-middle, IHC (OCN)-right) in PαS cells (*n* = 5). Scale bar = 100 μm, * *p* < 0.05, ** *p* < 0.01. Error bars indicate the standard error.

To obtain sufficient primary PαS cells, mice at three age points were tested (2–3 days, 8 weeks and 10 weeks old). The 2–3 days-old mice could offer 21.34 ± 0.75 ×10^4^ cells/mouse, which yielded the highest average cell extraction (Figure S1). As shown in Figure 2a,b, the intracellular ROS level increased nearly 4-fold when cells were cultured from in vivo (passage Fresh) to in vitro (passage P0). *P16* expression exhibited a nearly 4000-fold increase in adherent culture (passage P0), with a greater than 2.5-fold increase of *P53* compared to Fresh (Figure 2c). Consistently, the expression of the anti-aging gene *Sirt1* in P0 cells was 0.67-fold that in Fresh (Figure 2c).

To further demonstrate the differences between primary and cultured cells, SASP-associated genes were detected by qRT-PCR, in which *IL-8, Gm-Csf, Sfrp2, Wnt16b*, and *Ereg* were all upregulated from 1.3 to over nine times in cultured cells, statistical analysis indicated that these differences were all remarkable (Figure 2d, *p* < 0.05).

Telomere-related changes are an additional indicator of cellular senescence. Telomerase activity decreased from 7.215 ± 0.204 U/gprot (Fresh) to 5.246 ± 0.127 U/gprot (P0), indicating that the telomerase activity of cells significantly reduced; the average telomere length showed no significant difference (Figure 2e) between freshly isolated and P0 cells.

We further used nude mice transplantation to study the in vivo differentiation potential of these cells. H&E and Masson’s trichrome staining showed that P0 cells generated less bone-like tissue (1.58 ± 0.24%) than did Fresh cells (3.53 ± 0.30%) (Figure 2f). Osteoblast marker (osteocalcin, OCN) expression observed through IHC staining (Figure 2f) was lower in P0 cells. Thus, in vitro, the culture likely impaired the osteogenic differentiation of BMSCs.

### 2.2. Cellular Senescence Increased as Cells Were Cultured In Vitro

**Figure 3:**
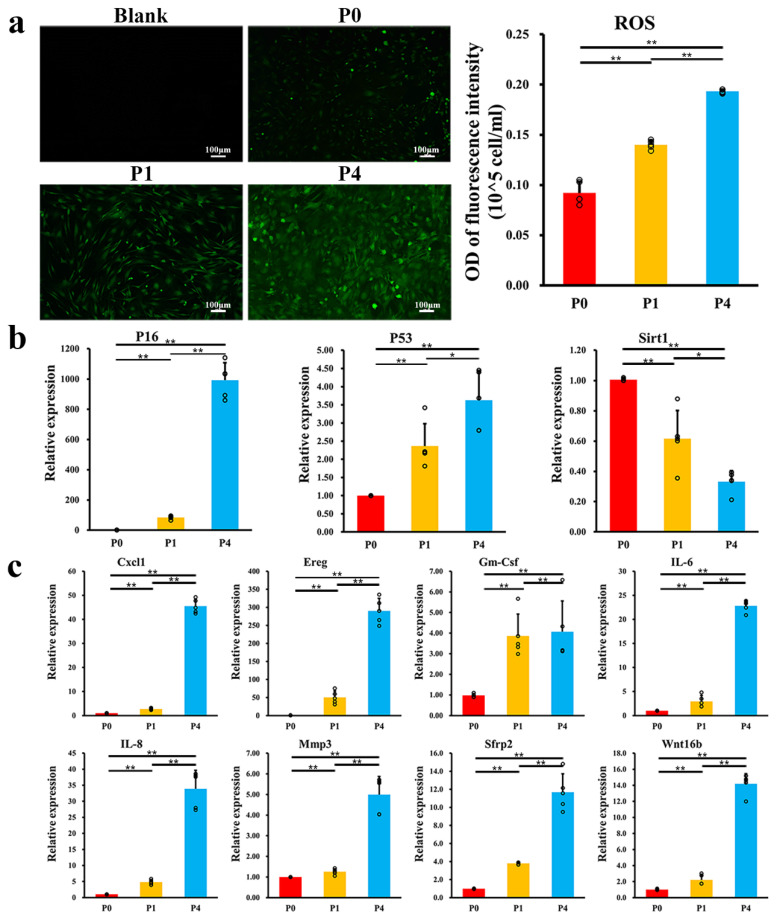
ROS and genes changed as PαS cells were cultured with the increase of cell passage (**a**) Intracellular total ROS staining and quantitative analysis (*n* = 5) of PαS cells with the increase of cell passages. (**b**) The expression of senescence-associated genes (*P16*-left, *P53*-middle, *Sirt1*-right) with the increase of cell passages (*n* = 5). (**c**) The mRNA levels of SASP genes (*IL-6, IL-8, Gm-Csf, Sfrp2, Ereg*, *Wnt16b, Mmp3* and *Cxcl1*) with the increase of cell passages (*n* = 5). Scale bar = 100 μm, * *p* < 0.05, ** *p* < 0.01. Error bars indicate the standard error.

We observed a sustained increase in ROS levels (Figure 3a) during in vitro culture. The expression of *P16* increased to 900-fold in P4 cells with a greater than 3.6-fold increase in *P53*, while *Sirt1* decreased to one-third of that in P0 cells (Figure 3b). We selected the SASP-associated genes *IL-6, IL-8, Gm-Csf, Sfrp2, Ereg, Wnt16b, Mmp3, and Cxcl1* (Figure 3c). All the SASP-associated genes were upregulated after passages of culture, especially between passage P1 and passage P4, in which *Ereg* increased up to 290 times.

**Figure 4:**
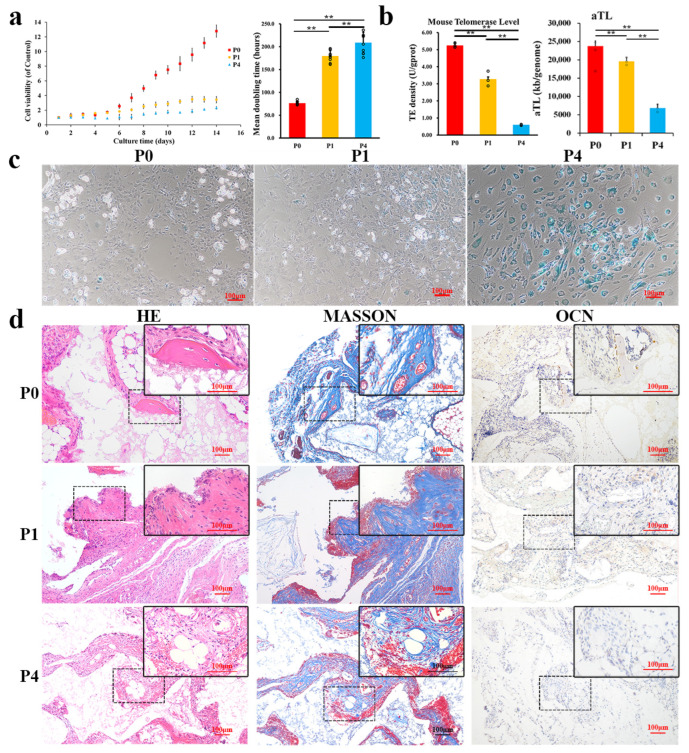
Senescence increased with the increase of cell passage (**a**) Cell proliferation ability (cell count-left, double time-right) changes with the increase of cell passages (*n* = 5). (**b**) Telomerase activity and average telomere length decreased with the increase of cell passages (*n* = 3). (**c**) The stain of senescence-association β-galactosidase increased with the increase of cell passages. (**d**) The ectopic osteogenesis capacity with the increase of cell passages (HE-left, Masson-middle, IHC (OCN)-right). Scale bar = 100 μm, ** *p* < 0.01. Error bars indicate the standard error.

Cell viability was detected using a cell proliferation and CCK-8. Compared with that for P1 and P4, the cell proliferation curve of P0 cells increased faster, and the platform stage of cell proliferation in the test cycle (14 days) was not observed (Figure 4a). The cell doubling time of P0 cells (76.34 ± 3.63 h) was significantly shorter than that of the remaining groups (P1-179.70 ± 12.27 h, P4-208.86 ± 22.89 h).

The average telomere length (aTL) of cells decreased from P0 (23721.97 ± 1476.02) to P4 (6821.88 ± 1022.80), with significant differences among the groups (Figure 4b), suggesting that aTL gradually decreased with the passage. The telomerase activity test showed that telomerase activity in P0 (5.246 ± 0.127 U/gprot) was significantly higher than that in P1 (3.274 ± 0.434 U/gprot) and P4 (0.602 ± 0.036 U/gprot). These data suggested that the telomerase activity in cells continued to decrease during in vitro culture (Figure 4b).

β-galactosidase staining was chosen as one of the hallmarks of cell senescence [16,29]. All groups showed positive staining (Figure 4c). However, fewer positive cells appeared in P0, while the number of positive cells was slightly higher in P1 and significantly increased in P4. These results indicated that the positive staining increased with the increase of passage, and the proportion of cells involved gradually increased.

The ectopic osteogenic capacity of the cells decreased as they were passaged in vitro (Figure 4d). Lamellar bone formation was observed only in P0. Meanwhile, Masson’s trichrome showed that the reticular and collagen fiber content decreased as cells were passaged. IHC staining showed that the OCN expression in passage P0 was also higher than that in P1 and P4.

### 2.3. Transcriptomic Differences between Fresh and Cultured Cells

**Figure 5:**
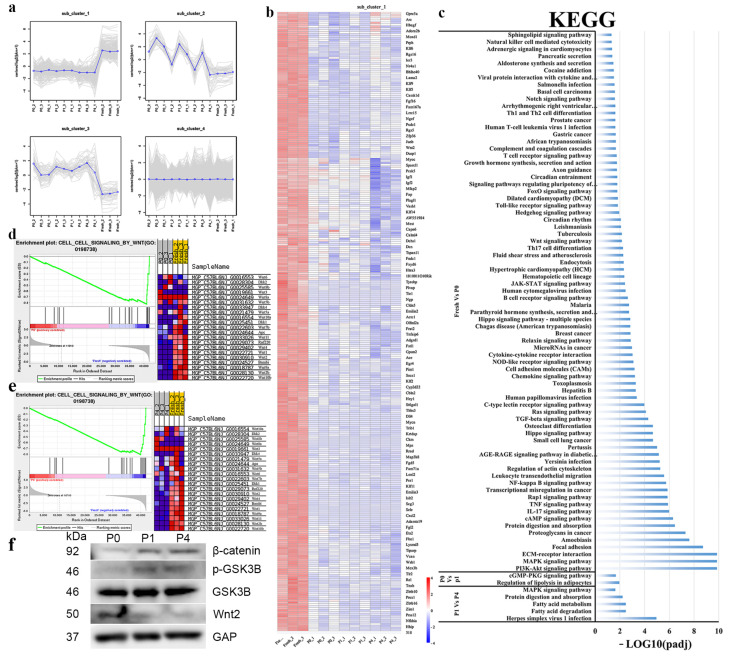
Transcriptional changes when PαS cells were cultured (**a**) Differential genes were sub-grouped into four sub-clusters. (**b**) Heatmap of sub_cluster_1. (**c**) KEGG enrichment analysis of decreased genes in different passages. (**d**) Gene Set Enrichment Analysis (GSEA) was determined to show the difference in the Wnt-related Gene Set between Fresh and P0 cells. (**e**) GSEA was determined to show the difference in the Wnt-related Gene Set between Fresh and P4 cells. (**f**) β-catenin, pGSK3B, GSK3B and Wnt2 protein expressions were determined by Western blot.

To identify the mechanisms underlying our observations, we detected the transcriptomic changes between Fresh and cultured cells by RNA-seq. Differentially expressed genes could be grouped into 4 subgroups: one for genes that were downregulated in other groups compared to the fresh group; one for genes whose expression increased during the transition from in vivo to in vitro; one for genes that increased between fresh and P0 but decreased at later passages; and the last one with the gene that showed no change among the groups (Figure 5a). We focused on the genes that decreased in cultured cells compared to Fresh cells (Figure 5a sub_cluster_1). The heatmap of transcription factor-related genes in sub_cluster_1 was drawn (Figure 5b). *Peg3, Gils2* and *Zim1* decreased significantly from Fresh to P4. These were therefore selected as potential target genes.

The significantly enriched KEGG pathways between the groups were used to identify pathways that were downregulated from Fresh to P0, P0 to P1, or P1 to P4 (Figure 5c). There were 76 pathways with changes between Fresh and P0, 2 pathways between P0 and P1, and 5 pathways between P1 and P4. *Zim1* was found in herpes simplex virus 1 infection, as shown in Figure 5c.

The Wnt signaling pathway changed significantly as cells were cultured. GSEA analysis was used to study Wnt-pathway-related gene sets between the Fresh-P0 (Figure 5d) and Fresh-P4 (Figure 5e) groups. The broken line plot of the GESA-enriched gene enrichment score showed high peak distribution at both ends, indicating that *Wnt2* was significantly enriched in the Fresh group. The rank value distribution plot for the genes also showed a higher degree of association with the phenotype for this gene set. Wnt-related genes were extracted and individually plotted using the GSEA Heatmap analysis tool, and the results showed similar genes between the GESA heatmaps (Figure 5d,e). Western Bolt showed the expression of β-catenin and p-GSK3B increased as cells were passaged, while Wnt2 significantly decreased (Figure 5f).

### 2.4. Zim1 Played a Critical Role in Suppressing Cell Aging

**Figure 6:**
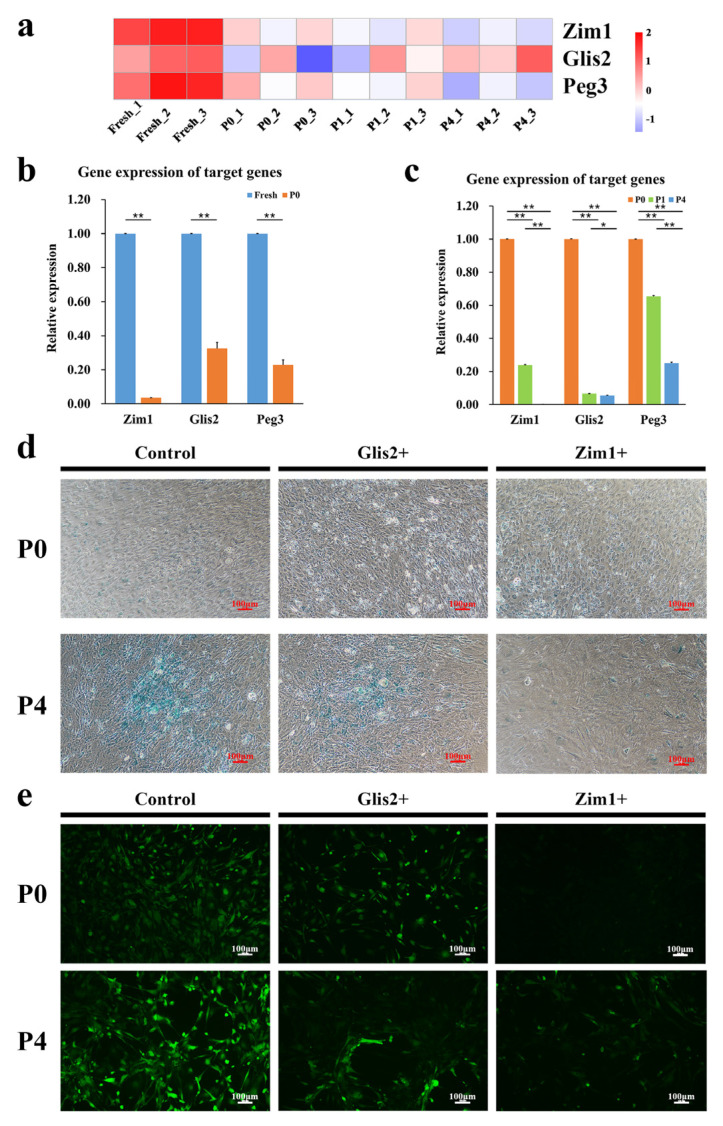
Target genes selection after RNA-seq analysis (**a**) Transcriptomic analysis was used to determine the expression level of *Peg3, Glis2* and *Zim1* in different passages. (**b**) Target gene levels between Fresh and P0 (*n* = 3). (**c**) Target gene levels among culture passages (*n* = 3). (**d**) The stain of senescence-association β-galactosidase with the overexpression of genes. (**e**) ROS staining of PαS cells with the overexpression of genes at P0 and P4. Control cells were cultured with an empty plasmid, Glis2+-cells were cultured with *Glis2* overexpression plasmid, Zim1+-cells were cultured with *Zim1* overexpression plasmid, Scale bar = 100 μm, * *p* < 0.05, ** *p* < 0.01. Error bars indicate the standard error.

Based on the RNA-seq results, we focused on several transcription factors, which were significantly downregulated or lost when the cells were cultured in vitro (Figure 6a). As shown in Figure 6b,c, the mRNA level of *Peg3*, *Gils2* and *Zim1* declined to less than one-third from Fresh to P0, less than one-fourth from P0 to P4, especially in *Gils2* and *Zim1*, which confirmed the accuracy of the RNA-seq analysis.

**Figure 7:**
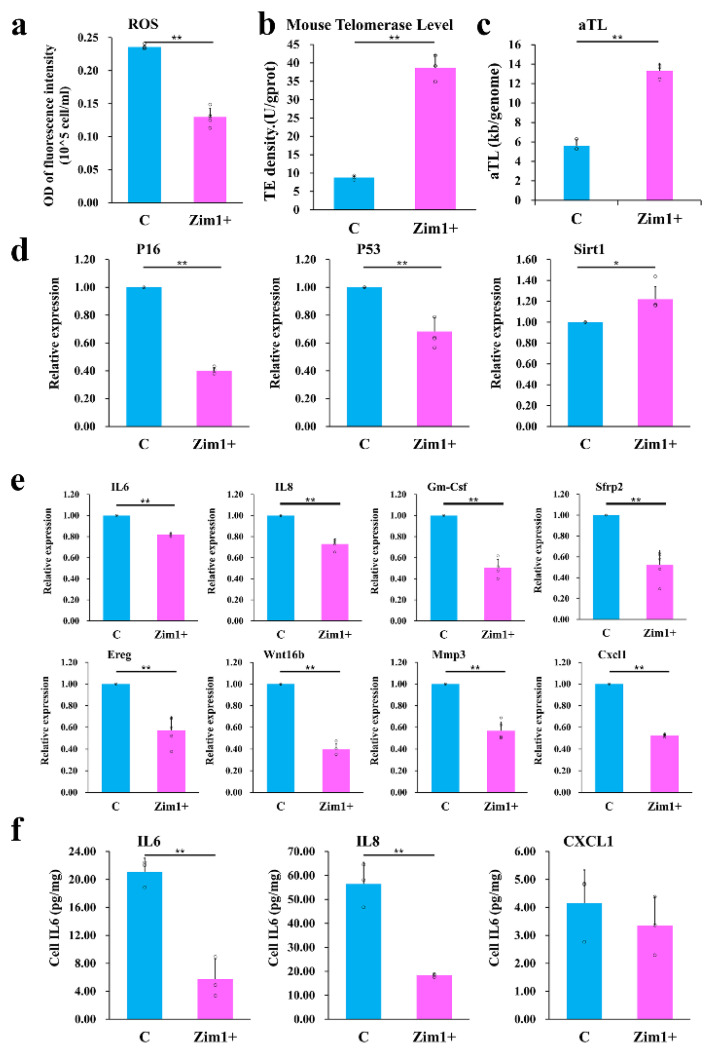
Zim1 played a critical role in suppressing cell aging (**a**) ROS quantitative analysis of PαS cells with the overexpression of *Zim1* at P4 (*n* = 5). (**b**) Telomerase activity of *Zim1* overexpressed at P4 (*n* = 3). (**c**) Average telomere length of *Zim1* overexpressed at P4 (*n* = 3). (**d**) The expression of senescence-associated genes (*P16*-left, *P53*-middle, *Sirt1*-right) with expression of *Zim1* (*n* = 5). (**e**) The mRNA levels of SASP genes (*IL-6, IL-8, Gm-Csf, Sfrp2, Ereg*, *Wnt16b, Mmp3* and *Cxcl1*) of *Zim1* overexpressed at P4 (*n* = 5). (**f**) Cell IL6, IL8 and CXCL1 levels of *Zim1* overexpressed at P4 (*n* = 3). C, cells were cultured with an empty plasmid, Zim1+, cells were cultured with *Zim1* overexpression plasmid, * *p* < 0.05, ** *p* < 0.01. Error bars indicate the standard error.

To confirm the potential role of these transcription factors, overexpression plasmids for *Gils2* and *Zim1* were constructed (Figure S2a), and senescence-association β-galactosidase staining was performed (Figure 6d). The results suggested *Zim1* as the target gene. In the ROS test, cells with *Zim1* overexpression showed significantly lower fluorescence signals than did the controls (Figure 6e). Quantitative analysis of ROS showed that ROS levels in cells overexpressing *Zim1* were nearly half of those in the control group (Figure 7a).

Telomerase activity assays showed that *Zim1* overexpression caused a 4.4-fold increase in the activity of the telomerase (Figure 7b). The mean telomere length significantly increased from 5.62 ± 0.59 KB/genome (empty plasmid) to 13.32 ± 0.78 KB/genome (*Zim1* overexpression group; Figure 7c).

Reverse transcription-quantitative PCR(RT-qPCR) showed that higher expression of *Zim1* could halve the level of *P16* and *P53* expression (Figure 7d). *SIRT1* levels of the *Zim1* overexpression group reached 1.2 times that of the control group (*p* < 0.05, Figure 7d), suggesting a slowing of cellular senescence. Further, SASP-related genes were also downregulated when *Zim1* was overexpressed in P4 cells (Figure 7e). ELISA tests also showed that the cell IL6, IL8 and CXCL1 levels decreased as *Zim1* was overexpressed (Figure 7f).

**Figure 8:**
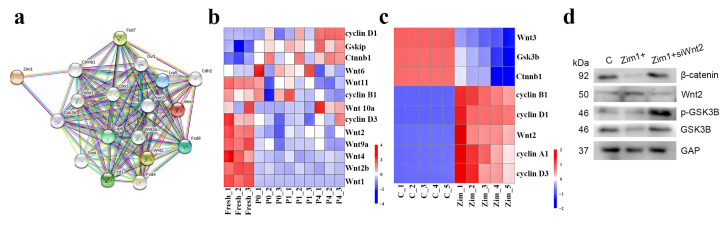
Cellular mechanism of *Zim1* in suppressing cell aging (**a**) Protein Network of ZIM1 and WNT2/WNT3 Interactions Predicted by STRING database. (**b**) Expression of Wnt pathway-related gene in RNA-seq. (**c**) Wnt pathway changes after overexpression of Zim1 (*n* = 5). (**d**) β-catenin, p-GSK3B and GSK3B protein expressions were determined after *Zim1* was overexpressed by Western blot.

Because RNA-seq analysis showed that the Wnt pathway changed during cell adherent culture and passage (Figure 5a,b), the STRING database was used to predict and analyze the correlation between ZIM1 and WntT2 proteins; the results suggested that *Zim1* could cause changes in *Wnt2* by interaction with *Gsk3b* and *Ctnnb1* (Figure 8a). In order to verify whether *Zim1* regulated MSCs anti-aging through Wnt-pathway, we found that *Zim1* overexpression could efficiently increase the expression of *Wnt2, cyclin A1, cyclin B1, cyclin D1* and *cyclin D3*, block the expression of *Gsk3b*, *Ctnnb1* and *Wnt3* (Figure 8b). Then, we performed siRNA-mediated knockdown of *Wnt2* in *Zim1* overexpressing cells. As shown in Figure 8c,d, Wnt2 deficiency could reverse the changes of β-catenin, p-GSK3B and GSK3B. Therefore, *Zim1* functioned as a positive regulator of MSC anti-aging through modulating Wnt2 signaling activation. Heatmaps of Wnt-pathway-related gene expression in the RNA-seq results are shown in Figure 8b and were reversed by *Zim1* overexpression (Figure 8c,d).

### 2.5. Zim1 Overexpression Combined with GelMA Has a Synergistic Effect on Anti-Aging

**Figure 9:**
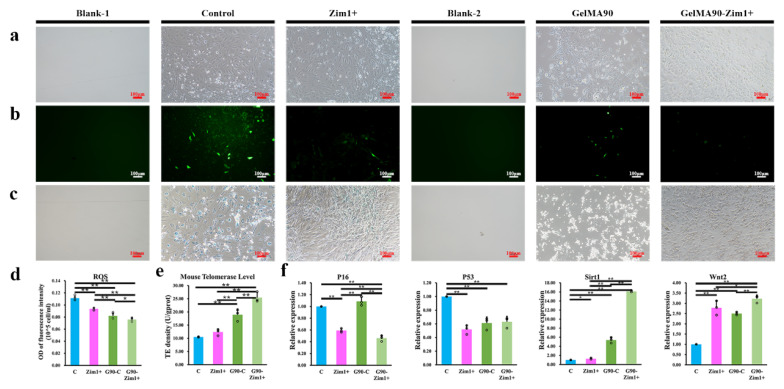
Senescence decreased GelMA90 combined with *Zim1* overexpression was applied in PαS cells (**a**) Cell morphology of PαS cells at P4. (**b**) ROS staining of PαS cells at P4. (**c**) The stain of senescence-association β-galactosidase decreased with the overexpression of *Zim1* at P4. (**d**) ROS quantitative analysis of PαS cells among the groups (*n* = 5). (**e**) Telomerase activities changed among the groups (*n* = 3). (**f**) The mRNA levels of genes (*P16*, *P53*, *Sirt1* and *Wnt2*) with the application of GelMA90 and *Zim1* overexpression (*n* = 3). Control, cells were cultured with empty plasmid; Zim1+, cells were cultured with *Zim1* overexpression plasmid; GelMA90, cells were cultured on GelMA90, Scale bar = 100 μm, * *p* < 0.05, ** *p* < 0.01. Error bars indicate the standard error.

Two kinds of substituted gelatin methacrylate (GelMA30 and GelMA90) were chosen, and GelMA90 showed better performance. As shown in Figure S2b, cells cultured on GelMA90 offered lower ROS fluorescence than that of GelMA30. There were few SA-β-Gal positive cells in GelMA30 and GelMA90, while the control group was contrary (Figure S2c). When the cell fusion degree reached about 80% on GelMA90, plasmid transfection was performed. Compared with the empty plasmid, cells transfected with *Zim1* overexpression plasmid were narrow and long, and the volume was slightly smaller than that of the controls. The cells on the surface of the GelMA90 hydrogel were slenderer and more uniform than the controls (Figure 9a).

ROS fluorescence intensity in cells overexpressing *Zim1* or cultured in GelMA90 was clearly lower (Figure 9b) than that in the control group. The ROS level was decreased by *Zim1* overexpression (0.094 ± 0.002) or cultured on GelMA90 (0.082 ± 0.005), with the extent decreased as they were combined together (0.076 ± 0.003, *p* < 0.05, shown in Figure 9d). SA-β-Gal staining of cells showed lighter staining positive in the *Zim1* overexpression group than in the remaining groups. After overexpression of *Zim1* on GelMA90, cells showed lighter staining than that in the group with only *Zim1* overexpression (Figure 9c). Telomerase activities were also increased from 10.51 ± 0.11 to 25.430 ± 1.98 as the combination of *Zim1* and GelMA90 was applied (Figure 9e).

RT-qPCR analysis of *P16* and *P53* showed that *Zim1* overexpression in GelMA90 hydrogel culture could reduce mRNA level (*P16* and *P53*) to about half of the control (*p* < 0.05, Figure 9f). Compared with the control group, *Sirt1* levels increased significantly in cells cultured on the surface of GelMA90 hydrogel (1.26 ± 0.16) and in cells overexpressing *Zim1* overexpressed (5.38 ± 0.62), the levels further increased when *Zim1* was overexpressed in GelMA90 hydrogel culture (16.09 ± 0.20, Figure 9d).

*Wnt2* expression in each group was also determined. As shown in Figure 5f, the *Wnt2* levels in the empty plasmid group were significantly lower than those in the other groups, and the GelMA90 group showed 2.5 times higher *Wnt2* levels than those in the control (*p* < 0.05). The *Wnt2* levels after GelMA90 hydrogel culture with *Zim1* overexpression (3.23 ± 0.18) were significantly higher than that in cells cultured in hydrogel alone (*p* < 0.05, Figure 9f).

## 3. Discussion

Aging is known as a universal, inherent and harmful process, appearing as the progressive loss of tissue and organ function, which result in the increasing of human pathologies [20]. Many studies have reported senescence in cellular cultures, while the state between internal and external is still rarely reported [16,30]. Meanwhile, our recent work pointed out that cultured MSCs may undergo the ‘active proliferation-differentiation-senescence’ processes, showing the importance of exploring cell senescence through cell culturing [31]. PαS cells, which can be efficiently purified by flow cytometry and used for pathophysiological studies as BMSCs, were pioneered in maximally mimicking the in vivo state of BMSCs at the time they were purified (named as Fresh) in this study [12,13]. The analysis of RNA-seq and cellular senescence assay expresses that the null hypothesis has been rejected. *Zim1*, reported highly expressed in the embryo, placenta and tip of the limb bud [32,33,34], was first found benefited to cell anti-aging. Meanwhile, hydrogels (GelMA) were also applied to study the effect of anti-cellular senescence, aiming to explore the possibility of combining hydrogels with genetic intervention.

The diverse manifestations of cell aging make it difficult to accurately define cell aging and exclude false positive aging caused by other factors by using a single aspect of detection [16,35,36]. Therefore, this study chose a variety of aging-related indicators to explore the change in aging status in cells. Results showed that cell cycle arrest-related pathways were activated [37], ROS levels rose immediately [38], the level of SASP-related genes increased [39], SA-β-Gal activities increased [16], and telomerase and aTL activities decreased [16], suggesting cellular senescence occurred at the beginning of culture. When PαS cells were passaged in vitro, data showed a similar trend to the above process, suggesting that cell senescence is continuous during culture.

RNA-seq results indicated that several genes were potential candidates to prevent or reverse cell aging. Of these, we tested three genes (*Peg3*, *Gils2,* and *Zim1*) and found that *Zim1* decreased more sharply in gene expression as cells were cultured (Figure 6b,c). Meanwhile, *Zim1* performed better in SA-β-Gal staining and ROS level (Figure 6d,e). Takikawa et al. obtained multiple iPS clones from mouse somatic cells and found that the parental-specific expression of *Zim1* was variably lost in the iPS clones [40]. Cattanach et al. contrasted the developmental fate of mice when *Peg3* or *Zim1* were deleted paternally or maternally and found that *Zim1* may play an important role during embryonic and neonatal mouse development [41]. Therefore, we finally focused on the zinc finger imprinted gene *Zim1*. With the overexpression of *Zim1,* the P16^Ink4a^-RB and P53/P21^CIP1^ pathways were inhibited, ROS and SASP gene levels decreased, telomerase activity increased, and SA-β-Gal activity was inhibited. To our knowledge, these results have not been reported to date.

Protein prediction by STRING database using ZIM1, WNT2, and WNT3 revealed that ZIM1 might act through GSK3B and CTNNB1 to link WNT2 or WNT3, thereby activating the expression of *Wnt2* while repressing *Wnt3*. RT-qPCR suggested that *Zim1* might affect the expression of *Wnt2* or *Wnt3* through *GSK-3β* and *Ctnnb1*. Western Bolt also showed that *Zim1* overexpression could reverse the changes in WNT2-related proteins during cell aging, with similar changes in GSK3B reported by Wang [42]. Researchers also confirm that WNTs and their receptors participate in cell proliferation, differentiation, migration, and patterning [42,43]. Shen et al. consider that WNT2 showed a trend of higher expression in the young group, and WNT2 ligand and downstream canonical WNT signals were repressed in senescent human cells [44,45], which showed a similar trend in this work. Shi also suggests that GSK3B activity is crucial for the SAHF formation following the downregulation of the Wnt pathway [46]. Feng also pointed out that GSK3B was upregulated by blocking WNT2 [47]. Bhavanasi et al. claimed that CTNNB1 enhanced the expression of GSK3B [48], which was similar to the RNA-seq results of this study. Taken together, *Zim1* might be a potential target for activating the Wnt2, downregulating the activity of GSK3B by reducing β-catenin, which is related to the maintenance of cells and prevention of cell aging.

With the recent interest in organoid research, the variety of hydrogel materials developed for cell culture has increased [49]. The development and improvement of GelMA hydrogel based on gelatin hydrogel has attracted much interest [21,26,29,50,51]. Gresham et al. reported GelMA might be helpful in anti-aging as fisetin was added to the culture medium, while fisetin treatment did not affect the senescent or osteogenic phenotype of MSCs alone [52]. Zhao showed that a 3D-printed three-layer gradient scaffold by GelMA could improve the chondrogenic differentiation of adipose-derived mesenchymal stem cells [53]. However, the effect of GelMA hydrogel cell culture on cell senescence has not been studied extensively. In this study, we determined the effect of GelMA hydrogel on cell senescence phenotypes and the effect of *Zim1* overexpression in GelMA hydrogel cultures. Cells cultured on GelMA were narrow and long, indicating lower senescence. When GelMA cultured cells overexpressed *Zim1*, the cell morphology became narrower and more uniform, expression of anti-aging-related genes increased, and ROS levels decreased. These changes suggested that *Zim1* overexpression and culture on GelMA had a synergistic effect against BMSC aging. RT-qPCR analysis also suggested that the zinc finger imprint gene (*Zim1*) and GelMA might inhibit the aging process of cells by increasing the expression of *Wnt2*. Thus, the zinc finger imprint gene and GelMA could have potential applications in the field of bone tissue engineering and cell expansion. Within the limitation of this study, the effects of the zinc finger imprint-related gene, zinc metabolism and GelMA on anti-aging in human BMSCs still need to be explored.

## 4. Materials and Methods

### 4.1. Mice and Cell Lines

C57BL/6 mice (2–3 days old, sex insensitive) and female BAL b/c mice (4–6 weeks old, 20–25 g) were obtained from Beijing Vital River Laboratory Animal Technology Co., Ltd (Beijing, China). Mice were housed in a specific pathogen-free environment with 12-h light/dark cycles. Experimental animals were treated in accordance with the Institutional Animal Guidelines approved by the Institutional Animal Care and Use Committee of Peking University (license no. LA2019019).

PαS cells were obtained and cultured as Morikawa and Diarmaid described [13]. In brief, cells were separated from bone fragments using collagenase, and PDGFR-α^+^SCA-1^+^CD45^-^TER119^-^ cells (passage Fresh) were purified and detected using a flow cytometer. Primary cultured cells (passage P0) were cultured in α-MEM (Gibco, Grand Island, New York, NY, USA) supplemented with 10% FBS (Gibco, Grand Island, New York, NY, USA) and 1% penicillin/streptomycin (Gibco, Grand Island, New York, NY, USA) at 37 °C with 5% CO_2_. The next passage was designated P1, and cells were cultured in serial passages to P4. Morphological observations were performed at passages P0, P1, and P4 under a microscope (Eclipse Ti2, Nikon, Tokyo, Japan).

GelMA (GelMAEFL-GM-90, EFL-Tech Co., Ltd., Suzhou, China) was also used to culture PαS cells (passage P4); cells were seeded on the hydrogel after gelatin directly reacted with methacryloyl in phosphate buffer, as Yue’s method [1,54].

### 4.2. Cell Proliferation and Doubling Time

CCK8 assay was performed to show the changes in cell proliferation at different passages, according to the manufacturer’s instructions (CCK8-500, Dojindi Labs, Kumamoto Prefecture, Japan). In brief, cells were seeded in a 96-well plate (1000 cells/well) and cultured for 1–14 days, and incubated with CCK8 reagent (10 μL/well) for 2 h. Standard curves were obtained by seeding 0–9000 cells/well in a 96-well plate under the same conditions of CCK8 incubation. The OD value at 450 nm was measured using a multimode microplate detection system (ELx800, Biotek, Winooski, VT, USA). Cell doubling times (T_D_) were calculated from the cell numbers obtained from the OD value and standard curves from individual experiments and for different passages using the equation:

T_D_ = (t × log2)/(logN_t_ − logN_0_) (1)

where N_0_ is the cell number at time 0, and N_t_ is the cell number at time t.

### 4.3. Reactive Oxygen Species (ROS) Test

ROS production was measured with an ROS assay kit (CA1410, Solarbio, Beijing, China) according to the manufacturer’s instructions. Briefly, cells were collected to 10^6^ cells/mL and incubated with the Opti-MEM medium (Gibco, Grand Island, New York, NY, USA) containing DCFH-DA for 20 min in the dark (37 °C). The relative fluorescence intensity was tested using the above microplate detection system. Relative ROS values were calculated versus the control. For intracellular total ROS staining, cells were treated with Opti-MEM medium with DCFH-DA for 20 min in the dark at 37 °C. Cellular fluorescence intensities were determined by fluorescence microscopy (Eclipse Ti2, Nikon, Tokyo, Japan).

### 4.4. RNA Collection and Reverse Transcription Quantitative PCR (RT-qPCR)

Total RNA extraction, determination of purity and concentration, and reverse transcription were performed as previously described [30]. SYBR Green Master Mix (11184ES08, Yeasen Biotechnology, Shanghai, China) and an RT-qPCR system (QuantStudio 3, Applied Biosystems, Waltham, MA, USA) were used to analyze mRNA levels. Glyceraldehyde-3-phosphate dehydrogenase (*Gapdh*) was chosen as the reference gene. The primer sequences of genes are shown in Table 1.

### 4.5. Telomerase Activity and Average Telomere Length

Total protein was extracted from RIPA lysate and detected using a BCA kit. Telomerase activities were detected according to the manufacturer’s instructions for the mouse telomerase ELISA kit (Shanghai Enzyme-linked Biotechnology Co., Ltd., Shanghai, China). The telomerase activities among the groups were normalized by total protein concentration.

Average telomere length (aTL) was detected, as described by O’Callaghan and Fenech [54]. DNA was extracted from the cells using a one-step mouse genotyping kit (PD101, Vazyme Biotech Co., Ltd., Nanjing, China). RT-qPCR was performed, and the 36B4 gene (single-copy gene) was selected as an internal control. The sequences of the primers are shown in Table 1.

### 4.6. SA-β-Gal Activity Staining

SA-β-gal activity was monitored by using the senescence-associated β-galactosidase staining kit (Beyotime Biotechnology, Shanghai, China) according to the instructions provided. Representative images of wells were photographed under a fluorescence microscope.

### 4.7. Expression Plasmid and siRNA Transfection

The *Glis2*-overexpressing plasmids (pcDNA-Glis2), *Zim1*-overexpressing plasmids (pcDNA-Zim1), and pcDNA3.1(+) empty control vector were constructed by Zhongmei Taihe Bio-Technology (Beijing, China). For WNT2, the inhibitory effect was achieved with siRNA (aca ccc aga ugu gau gcg ugc cau u) at 10 nM, according to Wang’s report [42]. These plasmids and siRNA were transfected into cells by using HighGene plus transfection reagent (ABclonal, Wuhan, China) according to the manufacturer’s instructions. Cells were collected, and the total RNA was extracted after 48 h of transfection.

### 4.8. RNA-Seq Analysis

Total RNA was collected and extracted using a TRIzol reagent. Raw reads of low quality were discarded with the removal of adaptors. The reference genome index was built using Hisat2 (v2.0.5), and paired-end clean reads were aligned according to the reference genome (mm10, GRCm38). The reads mapped to each gene were counted using FeatureCounts (v1.5.0-p3). Differential expression analysis between groups was performed using the DESeq2 (1.20.0, R package). Enriched biological processes and molecular functions, classified according to gene ontology (GO) terms, as well as KEGG pathways, were examined. Protein-protein interaction network (PPI) and visualization analysis were performed with Search Tool for the Retrieval of Interacting Genes from the STRING database (STRING v 11.5, http://string-db.org, accessed on 10 December, 2022) [55]. RT-qPCR was performed to confirm the expression levels of representative DEGs.

### 4.9. Evaluation of Ectopic Osteogenesis

Cells were trypsinized, resuspended and incubated with beta-tricalcium phosphate (β-TCP; Bicon, Boston, MA, USA) particles for 1 h at 37 °C. Then they were implanted into the subcutaneously dorsal space of BALB/c nude mice. Eight weeks later, implants were collected, fixed, decalcified, dehydrated and embedded in paraffin. Paraffin sections were stained with H&E and Masson’s trichrome together with an IHC assay.

### 4.10. Enzyme-Linked Immunosorbent Assay (ELISA)

Cell IL-6 (88-7064, Invitrogen, Carlsbad, CA, USA), IL-8 (EMC104, Neobioscience, Beijing, China) and CXCL1 (EK296/2, MultiSciences Biotech Co., Ltd., Hangzhou, China) levels were measured using a standard quantitative sandwich ELISA kit, according to the manufacturer’s instructions.

### 4.11. Western Blot

Total cellular protein was prepared in radioimmunoprecipitation assay (RIPA) buffer supplemented with 1% phosphatase and protease inhibitor cocktail (P002, NCM Biotech, Suzhou, China) on ice for 30 min. Then the lysates were centrifuged at 14,000 rpm, 4 °C for 20 min to collect the supernatants. Protein concentrations were measured by Pierce BCA protein assay kit (Thermo Fisher Scientific, Rockford, IL, USA). An equal amount of the protein extracts was separated on proper dodecyl sulfate, sodium salt-polyacrylamide gel electrophoresis and transferred to a polyvinylidene difluoride membrane (PVDF membrane, Millipore, Billerica, MA, USA). After blocking in 5% nonfat milk for 1 h, the membranes were incubated overnight at 4 °C with the primary antibodies. After rinsing in Tris-buffered saline-Tween 20 (TBST) 3 times, the membrane was incubated with IgG horseradish peroxidase-linked secondary antibody (1:10,000) for 1 h. Another 3 rinses in TBST were applied, and the electrochemiluminescence kit (CWBIO) was used to detect the protein bands. The following antibodies were used: abclone (Wuhan, China): Wnt2 (A13562); Proteintech (Chicago, IL, USA): β-catenin (51067-2-AP), GAPDH (60004-1-Ig); Huaxingbochuang Biotechnology (Beijing, China): pGSK3B (HX20130), GSK3B (HX20128); ZSGB-BIO (Beijing, China): Horseradish enzyme labeled goat anti-rabbit IgG (ZB-2301), Horseradish enzyme labeled goat anti-mouse IgG (ZB-2305).

### 4.12. Statistical Analyses

Data were shown as the mean ± SD and were analyzed using SPSS 26.0 software (IBM, New York, NY, USA). Statistical significance was determined by one-way ANOVA, and post hoc analysis among groups was applied. *p*-values < 0.05 were considered statistically significant.

## 5. Conclusions

In conclusion, the cellular senescence phenotypes occur in three important cell culture processes (from in vivo to first adherence in vitro, first adherence in vitro to the first passage, and serial passage in vitro). *Zim1* could be marked as a target gene to slow down cellular senescence and exhibited synergistic effects with biomaterials such as GelMA hydrogels. Our results suggest that C2H2-type zinc finger imprinting family genes and related proteins could be used as targets by the involvement of GSK3β and the Wnt pathway to prevent aging in cell cultures, tissue engineering, and other clinical stem cell applications.

## Figures and Tables

**Table 1:** Sequences of primers.

Name	Forward Primer (5′-3′)	Reverse Primer (5′-3′)
*m36B4*	ACTGGTCTAGGACCCGAGAAG	TCAATGGTGCCTCTGGAGATT
*TE-STD*	(TTAGGG)14	
*mTelo*	CGGTTTGTTTGGGTTTGGGTTTGGGTTTGGGTTTGGGTT	GGCTTGCCTTACCCTTACCCTTACCCTTACCCTTACCCT
*h36B4-STD*	CAGCAAGTGGGAAGGTGTAATCCGTCTCCACAGACAAGGCCAGGACTCGTTTGTACCCGTTGATGATAGAATGGG	
*h36b4*	CAGCAAGTGGGAAGGTGTAATCC	CCCATTCTATCATCAACGGGTACAA
*Gapdh*	CATCACTGCCACCCAGAAGACTG	ATGCCAGTGAGCTTCCCGTTCAG
*P16*	AACTCTTTCGGTCGTACCCC	GCGTGCTTGAGCTGAAGCTA
*P53*	GGAAGTCCTTTGCCCTGAACT	GTCTTCAGGTAGCTGGAGTGAG
*Sirt1*	GGAGCAGATTAGTAAGCGGCTTG	GTTACTGCCACAGGAACTAGAGG
*IL-8*	GGTGATATTCGAGACCATTTACTG	GCCAACAGTAGCCTTCACCCAT
*Gm-Csf*	GCTGCAGAATTTACTTTTCCTGGGC	TACCTCTTCATTCAACGTGACAGGC
*Sfrp2*	ACCCTTTGTAAAAATGACTTCGCACTG	ATTTCTTCAGGTCCCTTTCGGACAC
*Wnt16b*	CCCTCTTTGGCTATGAGCTGAG	GGTGGTTTCACAGGAACATTCGG
*Ereg*	CCCACCTTCTACAGGCAGTTATCAG	GGATCGTCTTCCATCTGAACTAAGGC
*Mmp3*	TGTCACTGGTACCAACCTATTC	TCTCAGGTTCCAGAGAGTTAGA
*Cxcl1*	TCCAGAGCTTGAAGGTGTTGCC	AACCAAGGGAGCTTCAGGGTCA
*Glis2*	TGGCCACTTTGTGTCACATGA	CGCTGACATAGGAGCCACTGT
*Zim1*	AGAACGAAAGCGCCATCCCA	GTGGCAGCGCTAGTTTGTCC
*Wnt2*	AGGATGCCAGAGCCCTGATGAA	CGCCTGTTTTCCTGAAGTCAGC
*Wnt3*	CCGCTCAGCTATGAACAAGCAC	AAGTCGCCAATGGCACGGAAGT
*Gsk3b*	GAGCCACTGATTACACGTCCAG	CCAACTGATCCACACCACTGTC
*Ccna1*	GCCCGACGTGGATGAGTTT	AGGAGGAATTGGTTGGTGGTT
*Ccnb1*	CTGAGCCTGAGCCTGAACCT	AGCCCCATCATCTGCGTCT
*Ccnd1*	GGGATGTGAGGGAAGAGGTGA	GCAGCGAAAACAACGTGAAA
*Ccnd3*	GCTCCAACCTTCTCAGTTGC	AGCTAAGCAGCAAGCAAAGC

## Data Availability

All data are included in the manuscript and supporting information. The datasets generated during and/or analyzed during the current study are available from the corresponding author upon reasonable request.

## Supplementary Materials

The following supporting information can be downloaded at: https://www.mdpi.com/article/10.3390/ijms24119766/s1.
