# Supplementary material for: ZIM1 Combined with Hydrogel Inhibits Senescence of Primary PαS Cells during In Vitro Expansion
Source: Int J Mol Sci. 2023 Jun 5;24(11):9766. doi: 10.3390/ijms24119766 (PMC10253596; doi:10.3390/ijms24119766)
Supplement: Supplementary file 1 [file ijms-24-09766-s001.zip › ijms-2438668-supplementary.pdf]

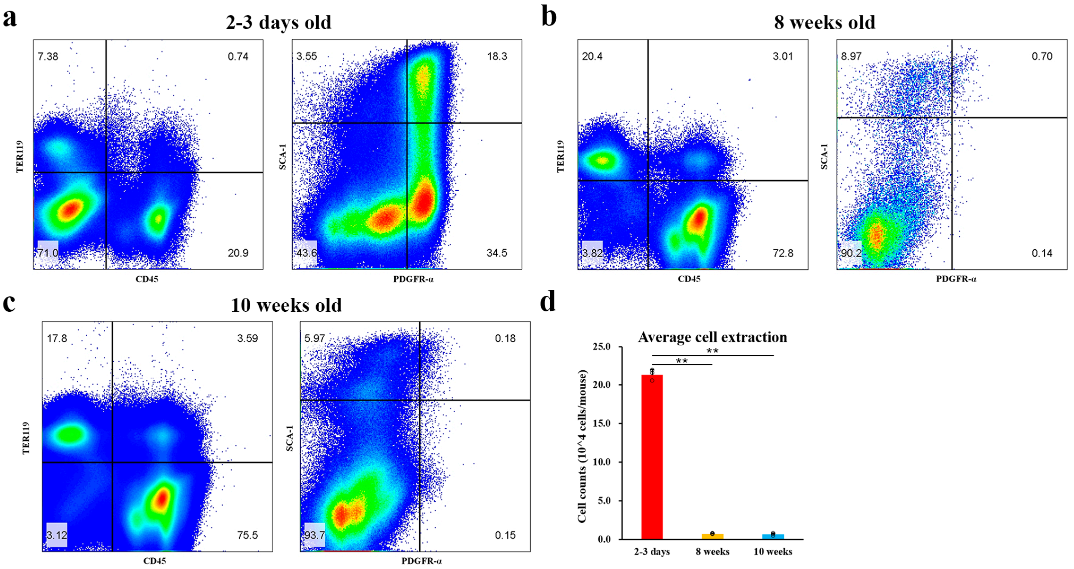

Figure S1. Cells isolated from total limb buds at postnatal days 2-3 (**a**), week 8 (**b**), and week 10 (**c**) were analyzed by flow cytometry. (d) Average PαS cells extraction of limb buds from different ages. ***p* < 0.01, Student's t test. Error bars indicate the standard error.

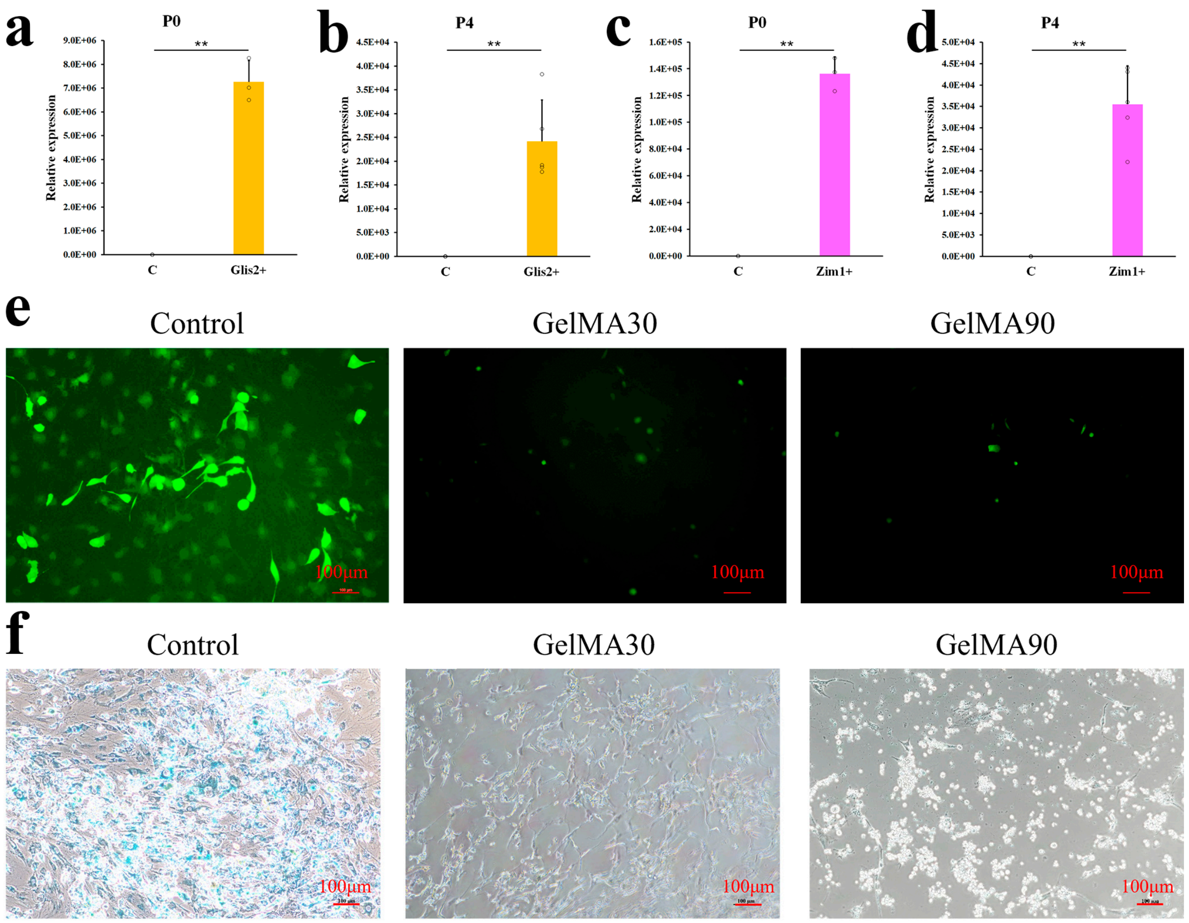

Figure S2 Selection of gene and GelMA (**a**-**d**) Changes in the expression levels of *Glis2*(**a**,**b**) and *Zim1*(**c**,**d**) after they were over-expressed during different cells passage (ac, P0, n = 3; bd, P4, n = 5). (**e**) ROS staining of PαS cells cultured on different GelMA. (**f**) The stain of senescence-association β-galactosidase

decreased as cells on GelMA30 or GelMA90. ***$p < 0.01$. Error bars indicate the standard error.
